# Development and validation of the Medical Student Scholar-Ideal Mentor Scale (MSS-IMS)

**DOI:** 10.1186/s12909-017-0969-1

**Published:** 2017-08-08

**Authors:** Stephen M. Sozio, Kitty S. Chan, Mary Catherine Beach

**Affiliations:** 1 Division of Nephrology, Department of Medicine, Johns Hopkins University School of Medicine, 301 Mason Lord Dr, Suite 2500, Baltimore, 21224 MD USA; 2 Welch Center for Prevention, Epidemiology, and Clinical Research, Johns Hopkins Medical Institutions, Baltimore, MD USA; 3 Department of Health Policy and Management, Johns Hopkins Bloomberg School of Public Health, Baltimore, MD USA; 4 Department of Health, Behavior, and Society, Johns Hopkins Bloomberg School of Public Health, Baltimore, MD USA; 5 Berman Institute of Bioethics, Johns Hopkins University, Baltimore, MD USA; 6 Division of General Internal Medicine, Department of Medicine, Johns Hopkins University School of Medicine, Baltimore, MD USA

**Keywords:** Mentorship, Medical Students, Scholarship

## Abstract

**Background:**

Programs encouraging medical student research such as Scholarly Concentrations (SC) are increasing nationally. However, there are few validated measures of mentoring quality tailored to medical students. We sought to modify and validate a mentoring scale for use in medical student research experiences.

**Methods:**

SC faculty created a scale evaluating how medical students assess mentors in the research setting. A validated graduate student scale of mentorship, the Ideal Mentor Scale, was modified by selecting 10 of the 34 original items most relevant for medical students and adding an item on project ownership. We administered this 11-item assessment to second year medical students in the Johns Hopkins University SC Program from 2011 to 2016, and performed exploratory factor analysis with oblique rotation to determine included items and subscales. We correlate overall mentoring quality scale and subscales with four student outcomes: ‘very satisfied’ with mentor, ‘more likely’ to do future research, project accepted at a national meeting, and highest SC faculty rating of student project.

**Results:**

Five hundred ninety-eight students responded (87% response rate). After factor analysis, we eliminated three items producing a final scale of overall mentoring quality (8 items, Cronbach’s alpha = 0.92) with three subscales: advocacy, responsiveness, and assistance. The overall mentoring quality scale was significantly associated with all four student outcomes, including mentor satisfaction: OR [(95% CI), *p*-value] 1.66 [(1.53–1.79), *p* < 0.001]; likelihood of future research: OR 1.06 [(1.03–1.09), *p* < 0.001]; abstract submission to national meetings: OR 1.05 [(1.02–1.08), *p* = 0.002]; and SC faculty rating of student projects: OR 1.08 [(1.03–1.14), *p* = 0.004]. Each subscale also correlated with overall mentor satisfaction, and the strongest relationship of each subscale was seen with ‘mentor advocacy.’

**Conclusions:**

Mentor quality can be reliably measured and associates with important medical student scholarly outcomes. Given the lack of tools, this scale can be used by other SC Programs to advance medical students’ scholarship.

**Electronic supplementary material:**

The online version of this article (doi:10.1186/s12909-017-0969-1) contains supplementary material, which is available to authorized users.

## Background

Medical education in the United States has required a balance of didactics, experiential learning, and development of lifelong learning. As part of this development, many programs have encouraged or required medical student scholarship. Formal curricula to navigate this research process has seen a rise in Scholarly Concentrations (SC) Programs, which allow students to understand subject areas of their interest in more depth beyond the traditional curriculum. While the exact structure of these SC Programs varies, common features include didactic training in scholarship, mentored experiences in research projects, and a final product to demonstrate program completion [1].

Even with these common features, SC Programs have struggled to evaluate the outcomes of the program. Outcomes typically reported include satisfaction with the program, publications and presentations achieved, and shaping of future career interests. The relationship between student experience and these outcomes has not been extensively explored; i.e. does a better student experience in a program lead to more research productivity or impact future careers? In terms of their experience, students in our course consistently rate the most important aspect of these programs as the opportunity to develop a mentoring relationship with a faculty member.

The concept of mentorship has been present since Greek mythology, and much of the prior work in this topic was in adult development and higher education [2]. With mentorship forming such a pivotal role in a medical student’s experience in scholarship, we need tools available to evaluate the quality of their mentorship experience. Mentorship in research and other scholarly activities is different from other types of mentorship that medical student’s experience [3] and mentoring medical students who may have a relatively brief amount of time to work on a project is different than mentoring doctoral students. Unfortunately, there are very few validated measures of mentorship quality as it relates to medical student experience in scholarship. The goal of our study was to develop and validate a measure of mentor quality specific for medical students who often have brief research experiences, the Medical Student Scholar-Ideal Mentor Scale (MSS-IMS).

## Methods

### SC program at Johns Hopkins

The SC Program at Johns Hopkins University School of Medicine began in 2009 as a required component of the M.D. curriculum, and is similar to other SC Programs across the country [4–9]. In the Johns Hopkins SC Program, students are guided to perform a scholarly project over their first two years in the preclinical curriculum, and prepare an abstract and in-person presentation of that project. In choosing a project, students are encouraged to think broadly about what they feel passionate about, what interests them, how they want to spend their summer between first and second year of medical school, and what field of medicine they wish to enter. During this process, they acquire skills for self-directed learning and identify options for pursuing a scholarly career in medicine. There are 5 areas of study (Concentrations) at Johns Hopkins: Basic Science; Clinical Research; History of Medicine; Medical Humanities and Bioethics; and Public Health and Community Service.

The program occupies 55.5 h in the curriculum over a period of approximately 18 months, typically in modular blocks over three days. There are four modules in the first year (December, February, March and May) and two modules in the second year (October and January). Students therefore must conduct almost all of the work on their scholarly project in their unscheduled time, and almost all of them do the bulk of the work in the summer between their first and second year. The course orientation is the only time when the entire class meets as a whole. Thereafter, for each of the subsequent modules, students meet within their Concentrations with their Concentrations faculty.

Throughout the curriculum, there are four basic written assignments (project proposal, summer progress report, project abstract, and poster or oral presentation), each with a preliminary and final version. SC faculty and students’ mentors provide written or oral individualized feedback to students on each assignment – both preliminary (formative feedback) and final (summative feedback) versions. Each student presents their scholarly project at Medical Student Research Symposium (MSRS), a partnership between the SC Program, the student organizing committee, and the Office of Student Affairs. All students (preclinical and clinical) are given the opportunity to present their scholarship, and are excused from their curricular activities for this afternoon event regardless of whether or not they are presenting. Awards are given to students through the Office of Student Affairs – these awards are not part of the SC Program but the Course Director assists the MSRS Organizing Committee in developing the judging process.

After each module, the Office of Curriculum sends out a questionnaire to students asking them how useful they found the modules, and whether they had any recommendations for improving it. We use these data each summer when we revise the curriculum for the following year. In addition, data about the SC Program are collected by the Course Director using student baseline and end-of-course evaluations, and student performance assessed with a structured faculty questionnaire. The Program has undergone slight modification over the years in response to student feedback; our lowest rated overall course evaluation was seen in the first year. However, the overall course goals, structure, and outcomes assessment has essentially remained unchanged over the years.

Potential faculty mentors are notified of course goals and asked, with each student, to sign a Mentor Agreement. Mentors are required to be faculty members, but can be either junior or senior faculty. Students continue in that mentor-mentee relationship over the entire 18-month SC Program duration; however, many of our students continue beyond that. Our most recent Mentor Information Sheet and Mentor Agreement describing expectations and goals are included in Additional file 1. Most of our mentors are experienced members of faculty at a mid-career level or greater, but some are at a more junior state (e.g. Assistant Professor).

### MSS -IMS scale item development

In 2009, a group of faculty teaching in the SC Program at Johns Hopkins searched the literature for scales about mentoring and found a paucity of available instruments. The best fit for our program was the Ideal Mentor Scale (IMS), a 32-item instrument designed to evaluate graduate student experience in mentorship with 3 subscales: Integrity (14 items), Guidance (10 items), and Relationship (10 items) [10]. Due to scale length and inclusion of items outside the focus of mentoring and/or research for the medical students in our program (e.g. keeps desk neat, talk to me about his personal problems, takes me out to dinner), we modified the IMS by selecting 10 items (3 from the integrity domain and 7 from the guidance domain). We also added one item which was critically important to research in our program but not included in the IMS (i.e., gives student sense of ownership over project). These 11 items are shown in Table 1. All items are rated on a 5-point Likert scale *(very satisfied, satisfied, neutral, dissatisfied, very dissatisfied)*.

**Table 1:** Candidate items used in Medical Student Scholar-Ideal Mentor Scale (MSS-IMS) questionnaire with original and new subscale assignments

Item	Original Sub-Scale	New Subscale
Inspires me by his or her example and words	Integrity	–^a^
Advocates for my needs and interests	Integrity	Advocacy
Gives proper credit to students	Integrity	Advocacy
Responds to emails and phone calls	Guidance	Responsiveness
Meets with me on a regular basis and when I need to	Guidance	Responsiveness
Provides information to help me understand the subject matter I am researching	Guidance	–^a^
Gives me a sense of ownership over the project	–(New)	Advocacy
Helps me plan a timetable for my research	Guidance	Assistance
Helps me prepare for a presentation	Guidance	Assistance
Helps me maintain a clear focus on my research objectives	Guidance	–^a^
Shows me how to employ relevant research techniques	Guidance	Assistance

^a^ *Items omitted after factor analysis*

### Study sample and data collection procedures

We administered the questionnaire to second-year medical students completing the SC Program at Johns Hopkins School of Medicine for six cohorts (2011–2016). At Johns Hopkins, MD-PhD students are not required to take the course and therefore we did not collect responses from students in a doctoral program. The end-of-course survey was administered through e*value, a commercially-available system that launches questionnaires. Students were given routine reminder emails weekly through that system. The survey contained the mentoring rating items as well as items about course satisfaction, dissemination plans for the project (if any) and the student’s own future plans.

In addition to student course evaluations, SC Program faculty rated each student’s performance in the course in the domains of class participation and project quality. At Johns Hopkins, each concentration has dedicated faculty who serve as student advisors but are not the primary mentor on the student project. Faculty evaluations were done in e*value with weekly reminders.

### Measurement of student outcomes

We assessed multiple student outcomes as a result of the SC Program, including:

- *Overall satisfaction with mentor*: In addition to the 11 mentor experience items in the end-of-course questionnaire, we also asked students to rate their overall satisfaction with their mentor on 5-point scale. For the purposes of analysis, we dichotomized this rating to compare the highest category (% very satisfied) with all other responses.
- *Likelihood of future research*: We asked students to report on the end-of-course questionnaire whether they were more or less likely (or the same) to pursue future scholarship as the result of their experience.
- *Abstract submitted to national/international meeting:* At the end of the course, students reported on whether or not they had submitted an abstract to a national or international meeting.
- *Faculty rating of student project:* We dichotomized the faculty ratings of student projects as those that SC Program faculty considered ‘excellent’ on all 5 criteria (importance of project, clarity of project presentation and goals, quality of design and methods, project organization, appropriateness of conclusions) with all those who had one or more criteria rated as less than excellent.

### Psychometric evaluation

#### Exploratory factor analysis

We first examined frequencies of student responses to the mentor scale items. Then following standard methods, [11] we produced a matrix of the correlations among all the items and conducted exploratory factor analysis to determine the number of relevant mentoring subdomains. We used oblique (promax) rotation, given the expectation of correlated factors for subdomains of mentoring quality, to identify potential subscales. Items that formed an interpretable factor and had factor loading ≥0.40 with the factor are included in a subscale. We eliminated three items that were highly correlated with all other items, or loaded onto more than one factor without providing additional important concepts, thus forming our final overall scale of mentoring quality, the MSS-IMS.

#### Scale validation: reliability and construct validity

To assess internal consistency reliability, we calculated Cronbach α for the overall MSS-IMS and subscales. In order to assess construct validity, we conducted logistic regression analyses to evaluate the association between overall mentor satisfaction with scores from the overall MSS-IMS and subscales. We also conducted logistic regression analyses to evaluate the association between the mentoring quality scales and the additional student outcomes (likelihood of future research in career, submission of abstract to national/international meeting, and highest faculty rating of project). We expect higher ratings for mentoring quality to be associated with greater satisfaction and better outcomes. For the analyses with mentor subscales, we included all the subscales in the model together to understand the unique contribution of each subscale.

Finally, we examined trends in response by exploring the relationship of both program year and Concentration selection on each mentor scale item using contingency tables and chi-square testing. All data were analyzed with STATA SE 14 (College Station, TX). A *p*-value <0.05 was considered significant.

## Results

### Study sample

Of the 685 second-year medical students in the SC Program from 2011 to 2016, 598 responded to the end-of-course questionnaire (87% response rate). Students answering the questionnaire represented the five concentrations at Johns Hopkins: Basic Science (*n* = 67, 11%), Clinical Research (*n* = 273, 46%), History of Medicine (*n* = 42, 7%), Ethics and the Art of Medicine (*n* = 44, 7%), and Public Health (*n* = 172, 29%).

Most students (69%) reported the highest level of overall satisfaction with their mentor, and 92% were satisfied or very satisfied. Fewer than half (44%) reported that they were more likely to do research in the future, and fewer than half (38%) reported that they had submitted an abstract by the end of the course. A small minority (13%) had the highest faculty rating of their project.

### Development of mentor scale and subscales

Across the 11 items on the mentor scale, students reported highest rating of satisfaction between 56 and 75%, and reported being ‘satisfied’ or ‘very satisfied’ between 82 and 94% (Table 2). Items receiving lowest satisfaction rating were in planning a timetable, employing research techniques, and preparing a presentation. The item with highest satisfaction rating was in project ownership. When examining trends in responses, there was no significant difference in distribution of the 11 mentor scale responses across years in the program or across Concentrations (*p* > 0.05 for all responses).

**Table 2:** Sub-Scales, items and factor loadings for items in overall Medical Student Scholar-Ideal Mentor Scale (MSS-IMS) and each subscale

	Sub-scale	Item	n (%) “Very Satisfied”	n (%) “Satisfied”	Factor Loadings^a^	
					Total Scale	Subscale
Overall MSS-IMS 8 items α = 0.921 Mean(SD) = 35.5 (6.3) Range 0–40 259 (43%) with highest score (40)	Mentor Advocacy 3 items α = 0.869 Mean (SD) = 13.8 (2.2) Range 0–15	Gives proper credit to students	436 (73%)	120 (20%)	0.765	0.645
		Advocates for my needs and interests	404 (68%)	136 (23%)	0.829	0.692
		Gives me a sense of ownership over the project	446 (75%)	116 (19%)	0.786	0.723
	Mentor Responsiveness 2 items α = 0.856 Mean (SD) = 9.0 (1.6) Range 0–10	Responds to emails and phone calls	411 (69%)	132 (22%)	0.803	0.535
		Meets with me on a regular basis and when I need to	400 (67%)	138 (23%)	0.809	0.540
	Mentor Assistance 3 items α = 0.858 Mean (SD) = 12.7 (3.1) Range 0–15	Helps me plan a timetable for my research	355 (59%)	140 (23%)	0.815	0.400
		Shows me how to employ relevant research techniques	332 (56%)	162 (27%)	0.776	0.705
		Helps me prepare for a presentation	341 (57%)	150 (25%)	0.715	0.756

^a^Factor analysis reported is based on the “very satisfied” outcome. Subscale factor loading from oblique rotation with three factors

After factor analysis, we produced a final scale of overall mentoring quality (MSS-IMS, 8 items, see Additional file 2) with three subscales: 1) advocacy (putting student first and giving sense of ownership, 3 items, alpha = 0.87), 2) responsiveness (availability for meetings and email, 2 items, alpha = 0.86), and 3) assistance (teaching specific skills, 3 items, alpha = 0.86). Grouping ‘satisfied’ and ‘very satisfied’ responses produced similar findings in correlations and factor analysis (data not shown). The items, subscales and factor loadings in overall mentoring quality and each subscale are shown in Table 2.

### Reliability and validity of mentor scale and subscales

The overall MSS-IMS (α = 0.92) and the three subscales (α > 0.85) were reliable. Association of the overall MSS-IMS with each dichotomous student outcome is shown in Table 3. The overall MSS-IMS and all of the mentor quality subscales were associated with student overall satisfaction with mentor in the expected direction. In addition, the overall MSS-IMS was significantly associated with all student outcome measures: likelihood of future scholarship, submission of abstract to national/international meeting, and highest faculty rating of project. The mentor advocacy subscale was significantly associated with being more likely to do future research and with having the highest faculty rating of project, whereas the other subscales were not significantly associated with any additional outcomes.

**Table 3:** Associations of overall Medical Student Scholar-Ideal Mentor Scale (MSS-IMS) and subscales with student and faculty-reported scholarly outcomes

	‘Very Satisfied’ with mentor OR (95% CI)	More likely to do future research OR (95% CI)	Abstract submitted National/International meeting OR (95% CI)	Highest faculty rating of project OR (95% CI)
Overall MSS-IMS	1.66 (1.53–1.79) *p* < 0.001	1.06 (1.03–1.09) *p* < 0.001	1.05 (1.02–1.08) *p* = 0.002	1.08 (1.03–1.14) *p* = 0.004
Mentor Advocacy^a^	2.06 (1.67–2.55) *p* < 0.001	1.22 (1.06–1.41) *p* = 0.005	1.05 (0.91–1.20) *p* = 0.507	1.29 (1.01–1.65) *p* = 0.041
Mentor Responsiveness^a^	1.92 (1.47–2.51) *p* < 0.001	0.96 (0.81–1.14) *p* = 0.646	0.98 (0.82–1.17) *p* = 0.803	0.84 (0.65–1.10) *p* = 0.215
Mentor Assistance^a^	1.37 (1.20–1.56) *p* < 0.001	1.01 (0.93–1.10) *p* = 0.738	1.09 (0.99–1.19) *p* = 0.073	1.10 (0.96–1.27) *p* = 0.166

^a^Adjusted for each of the other subscales

## Discussion

In this study, we adapted a validated graduate student scale of mentorship for medical students pursuing scholarship, creating the MSS-IMS. We found the adapted mentoring quality scale and subscales to have high internal consistency as well as construct validity. To our knowledge, this is the first such tool specifically addressing mentorship in medical student scholarly experiences, and can be implemented at other SC Programs nationwide.

We have provided a version of this tool in Additional file 2 to aid other investigators and SC Program faculty and administrators. This tool can be used to evaluate their own Program’s mentorship experience overall, and across individual or groups of mentors. In addition, the specific subscales of advocacy, responsiveness, and assistance can be assessed with this tool to aid in a more individualized Program’s development. While research experiences may differ across Programs, our diversity in scholarly experiences in our own Program allows this tool to be used across multiple disciplines.

Traditional mentorship in undergraduate medical education is multifocal, and includes such items as personal and professional development, and emotional support and encouragement [12]. A 2006 systematic review of mentorship for medical students identified 16 papers that described structured mentorship programs for medical students [13]. Only two articles specifically examined scholarship, and these two articles describe mentor activities but not quality of these experiences [14, 15]. A 2010 systematic review in PubMed identified 14 manuscripts describing medical student mentoring programs, with themes ranging from “career counseling, develop professionalism, increase students’ interest in research, and support them in their personal growth.” [3] For the articles describing research mentorship of medical students, outcomes reported included increased research skills, increased number of research papers, increased number of graduates in research careers, and an overall vision of how programs can improve research experience [16, 17]. However, only a description of the mentor experience and frequency of particular mentor activities were provided without assessment of the mentor experience, or the mentor’s impact. In addition, these studies do not describe an overall scale to use when assessing medical students’ experiences with mentors. This construct is important to measure so we can understand the impact that mentors have on student outcomes.

We were intrigued by the pattern of associations of the mentor quality subscales with student outcomes, with the mentor advocacy domain having the strongest association with future career intentions and a highly-rated project by the SC faculty. This suggests that students whose mentors are more generous (give them a sense of ownership, looks out for their interests, and gives them credit for their work) are able to find more passion for or investment in the work itself, leading to a greater interest in pursuing it further. There are several possible reasons why we might have found the association between mentor advocacy and faculty rating of the student projects. First, students who are given this particular kind of support may take more ownership, which could lead to a better project. Also, our SC Program explicitly values student ownership, which could mean that our SC faculty are rating the students based on their sense of how much ownership the student has taken, making them particularly enthusiastic about projects where that aspect of student engagement is more evident.

There are several limitations of this study. This is a single-institution study with replication limited to multiple years of our own institution’s students. However, we have several years experience with SC, and the detailed nature of the data make a multi-institution study difficult to perform. More granular characterization of themes around the mentorship experience was not available in our tool; however, we have added a free text comment field in our Additional file 2 for others using this tool. We also noticed that there was a trend between the subscale of mentor assistance and the student abstract outcome. It is possible that a more robust set of items for mentor assistance may produce a significant relationship with submitting abstracts to meetings. This is particularly relevant as mentor assistance was the lowest rated set of items for our students (82–83% of students rated as ‘satisfied’ or ‘very satisfied’.) Reasons for this relatively lower rating are unclear, but we will be exploring how to improve the delivery of these assistance aspects in our own Program. Adding mentor assistance items may improve our assessment and this scale, but would likely increase the length of the scale and the time required to complete it. Finally, the long-term impact of this mentoring relationship and scale in later years of medical school still needs analysis.

## Conclusions

In conclusion, the MSS-IMS is a valid and reliable tool to assess mentoring quality for medical students performing scholarship. Implementation of this tool in other SC Programs can help understand and advocate for their highest quality mentors.

## Additional files

Additional file 1: Mentor Information Sheet and Mentor Agreement. (PDF 2079 kb)

Additional file 2: Medical Student Scholar-Ideal Mentor Scale (MSS-IMS). (PDF 460 kb)

## Abbreviations

MSS-IMS: Medical Student Scholar-Ideal Mentor Scale
SC: Scholarly Concentrations

## References

[1] Bierer SB, Chen HC (2010). How to measure success: the impact of scholarly concentrations on students--a literature review. Acad Med.

[2] Levinson DJ, Darrow CN, Klein EB, Levinson MA, McKee B (1978). Seasons of a man’s life.

[3] Frei E, Stamm M, Buddeberg-Fischer B (2010). Mentoring programs for medical students--a review of the PubMed literature 2000-2008. BMC Med Educ.

[4] Langhammer CG, Garg K, Neubauer JA, Rosenthal S, Kinzy TG (2009). Medical student research exposure via a series of modular research programs. J Investig Med.

[5] Laskowitz DT, Drucker RP, Parsonnet J, Cross PC, Gesundheit N (2010). Engaging students in dedicated research and scholarship during medical school: the long-term experiences at Duke and Stanford. Acad Med.

[6] Rhyne RL (2000). A scholarly research requirement for medical students: the ultimate problem-based learning experience. Acad Med.

[7] Rosenblatt RA, Desnick L, Corrigan C, Keerbs A (2006). The evolution of a required research program for medical students at the University of Washington School of Medicine. Acad Med.

[8] Smith FG, Harasym PH, Mandin H, Lorscheider FL (2001). Development and evaluation of a Research Project Program for medical students at the University of Calgary Faculty of Medicine. Acad Med.

[9] Zier K, Stagnaro-Green A (2001). A multifaceted program to encourage medical students’ research. Acad Med.

[10] Rose GL (2003). Enhancement of mentor selection using the ideal mentor scale. Res High Educ.

[11] Nunnally JC, Bernstein IH (1994). Psychometric theory.

[12] Rose GL, Rukstalis MR, Schuckit MA (2005). Informal mentoring between faculty and medical students. Acad Med.

[13] Buddeberg-Fischer B, Herta KD (2006). Formal mentoring programmes for medical students and doctors--a review of the Medline literature. Medl Teach.

[14] Frishman WH (2001). Student research projects and theses: should they be a requirement for medical school graduation?. Heart Dis.

[15] Gonzales AO, Westfall J, Barley GE (1998). Promoting medical student involvement in primary care research. Fam Med.

[16] Keyser DJ, Lakoski JM, Lara-Cinisomo S, Schultz DJ, Williams VL, Zellers DF, Pincus HA (2008). Advancing institutional efforts to support research mentorship: a conceptual framework and self-assessment tool. Acad Med.

[17] Zier K, Friedman E, Smith L (2006). Supportive programs increase medical students’ research interest and productivity. J Investig Med.

