# Supplementary material for: Development and validation of the Medical Student Scholar-Ideal Mentor Scale (MSS-IMS)
Source: BMC Med Educ. 2017 Aug 8;17:132. doi: 10.1186/s12909-017-0969-1 (PMC5549328; doi:10.1186/s12909-017-0969-1)
Supplement: Supplementary file 1 — Mentor Information Sheet and Mentor Agreement. (PDF 2079 kb) [file 12909_2017_969_MOESM1_ESM.pdf]

Additional File 1: Mentor Information Sheet and Mentor Agreement

Scholarly Concentrations -Class of 2020 Information Sheet for Potential Mentors

What are the objectives for this course?

The overall goals of the SC program are to promote the student's intellectual curiosity, appreciation of scholarly inquiry, flexibility, passion for discovery, openness to new ideas, and the ability to work both independently and collaboratively. Each student in the SC program will be expected to engage in a mentored scholarly project in an area of individual interest, and to present their work in a written and oral format. Throughout the course, students will receive feedback from their mentor, concentration faculty and other students.

During Year 1, students will develop a research question and plan for a scholarly project. In consultation with their mentor and Concentration Leaders, students will develop a plan for completion of their scholarly project that uses the most appropriate methods. Concentration Leaders will also guide students towards a scope and timeline that ensures completion by the due date.

During Years 1-2, students will conduct an independent scholarly project. According to the scholarly plan and timeline, students will work independently on their projects. Work is typically done during the summer, with additional work required during the school year. Students are expected to present progress on their project in the Fall and make a final presentation early in 2018 at the Medical Research Day (expected date in Jan/Feb 2018)

What is the schedule for student projects?

**April 2017:**	**Preliminary Project Proposal Due**
**April 2017:**	**Submit proposal to IRB if applicable (new data collection or no existing IRB protocol)**
**June 2017:**	**Final Proposal Due**
**July-August 2017:**	**Full time work on Scholarly Project (approximately 7 weeks)**
**September 2017:**	**Progress Report Due**
**October 2017:**	**Preliminary Project Abstract Due**
**January-February 2018:**	**Final presentation of project at SC/Medical Student Research Day (MSRS)**

What are the mentor's responsibilities?

Primary mentors will most often be JHU faculty, although other individuals (e.g., post doctoral fellows, technicians, staff) may be involved in facilitating the student's project. **The primary faculty mentor has the following responsibilities**:

- Assist student in identifying a research or scholarly project
- Ensure that the student's involvement in the research project is approved by the IRB (if applicable). This means either adding the student to an existing IRB protocol or guide the student in writing an IRB application for their project (that has to be submitted by you).
- Agree to meet regularly during the academic year to plan the project and then weekly during the summer with the student and offer feedback and guidance on the progression of the project.
- Participate in the evaluation of the student's progress and provide feedback to the course instructors
- Suggest public forums where the student may present their research. The student will present at Medical Student Research Day in the SOM, but if other opportunities present themselves please suggest them to the student!

Scholarly Concentrations Class of 2020
Mentorship Agreement

SCHOLARLY CONCENTRATIONS PROGRAM
Johns Hopkins School of Medicine

Each student in the SC program will be expected to engage in a mentored scholarly project in an area of individual interest, complete assignments on time, and present their work in a written and oral format. Throughout the course, students will receive feedback from their mentor, concentration faculty and other students.

What is the schedule for student projects?

April 2017:	Preliminary Project Proposal Due
April 2017:	Submit proposal to IRB if applicable (new data collection or no existing IRB protocol)
June 2017:	Final Proposal Due
July-August 2017:	Full time work on Scholarly Project (approximately 7 weeks)
September 2017:	Progress Report Due
October 2017:	Preliminary Project Abstract Due
January-February 2018:	Final presentation of project at SC/Medical Student Research Symposium (MSRS)

What are the student mentee's responsibilities?

- Provide information to your faculty mentor on your interests, abilities, and goals.
- Work with your mentor to develop a scholarly project proposal and timeline; and ensure that your research project is approved by the IRB (if applicable).
- Schedule regular meetings with your mentor during the academic year; weekly during the summer (if possible); and then accordingly through MSRD.
- Update your mentor regularly on progress, including timely notification of unexpected delays or complications.
- Prepare drafts of course assignments (project proposal, abstract, and final presentation) for your mentor's review with adequate lead time to permit revision as necessary.

____
Student Name

____
Signature Date

What are the faculty* mentor's responsibilities?

- Assist student in identifying and refining a feasible research or scholarly project accordingly.
- Ensure that the student's involvement in the research project is approved by the IRB (if applicable) in a timely fashion so that the student can work over the summer.
- Review and provide feedback on course assignments (project proposal, abstract, and final presentation).
- Agree to meet with the student regularly during the academic year to plan the project; approximately weekly during the summer to offer feedback and guidance on the progression of the project; and then check in for follow-up and finalization of the project through MSRD in January.
- Support student by attending Medical Student Research Day
- Evaluate the student's progress and provide feedback, through Evalue, in January following MSRD.

____
Faculty Mentor Name

____
Signature

**Primary mentors will most often be JHU faculty, although other individuals (e.g., post doctoral fellows, technicians, staff) may be involved in facilitating the student's project.*
