# Supplementary material for: Development and validation of the Medical Student Scholar-Ideal Mentor Scale (MSS-IMS)
Source: BMC Med Educ. 2017 Aug 8;17:132. doi: 10.1186/s12909-017-0969-1 (PMC5549328; doi:10.1186/s12909-017-0969-1)
Supplement: Supplementary file 2 — Medical Student Scholar-Ideal Mentor Scale (MSS-IMS). (PDF 460 kb) [file 12909_2017_969_MOESM2_ESM.pdf]

Additional File 2: Medical Student Scholar-Ideal Mentor Scale (MSS-IMS)

EVALUATION OF MENTOR AND RESEARCH EXPERIENCE

SCHOLARLY CONCENTRATIONS PROGRAM
Johns Hopkins School of Medicine

Please rate your satisfaction with how your primary faculty research mentor relates to you in the following areas:

	Very Satisfied	Satisfied	Neutral	Dissatisfied	Very Dissatisfied	Not applicable
Gives proper credit to students	❍	❍	❍	❍	❍	❍
Advocates for my needs and interests	❍	❍	❍	❍	❍	❍
Responds to emails and phone calls	❍	❍	❍	❍	❍	❍
Meets with me on a regular basis and when I need to	❍	❍	❍	❍	❍	❍
Gives me a sense of ownership over the project	❍	❍	❍	❍	❍	❍
Helps me plan a timetable for my research	❍	❍	❍	❍	❍	❍
Helps me prepare for a presentation	❍	❍	❍	❍	❍	❍
Shows me how to employ relevant research techniques	❍	❍	❍	❍	❍	❍
Overall, how satisfied were you with your mentor?	❍	❍	❍	❍	❍	❍

Please provide any additional comments on your mentor or your mentorship experience:

____

____

____

____
